# A Mismatch between the Perceived Fighting Signal and Fighting Ability Reveals Survival and Physiological Costs for Bearers

**DOI:** 10.1371/journal.pone.0084571

**Published:** 2014-01-07

**Authors:** Isaac González-Santoyo, Daniel M. González-Tokman, Roberto E. Munguía-Steyer, Alex Córdoba-Aguilar

**Affiliations:** Departamento de Ecología Evolutiva, Instituto de Ecología, Universidad Nacional Autónoma de México, Ciudad Universitaria, Mexico D.F., México; University of Melbourne, Australia

## Abstract

Signals of fighting indicate an animal's intention to attack and so they serve to prevent costly aggressive encounters. However, according to theory, a signal that is different in design (i.e. a novel signal) but that fails to inform fighting intentions will result in negative fitness consequences for the bearer. In the present study we used males of the territorial damselfly *Hetaerina americana*, which have a red wing spot during territory defense that has evolved as a signal of fighting ability. By producing a novel signal (covering the red spot with blue ink) in territory owners, we investigated: a) the behavioral responses by conspecific males; b) survival cost and c) three physiological mediators of impaired survival: muscular fat reserves, muscle mass and immune ability. We predicted that males with the novel signal would be attacked more often by conspecifics as the former would fail to convey fighting ability and intentions adequately. This will result in lower survival and physiological condition for the novel signal bearers. We found that, compared to control males (males whose red spot was not changed), experimental males had reduced survival, were less able to hold a territory, and had a reduced muscle mass. It seems that spot modified males were not able to effectively communicate their territory tenancy, which may explain why they lost their defended sites. Our results provide support for theoretical models that a novel signal that fails to informing fighting ability may lead to a fitness cost for bearers.

## Introduction

Animal signals convey information to conspecifics and are frequently used to mediate aggressive conflicts. In particular, signals used to indicate fighting intentions are interpreted to communicate reliably an individual's intention to attack (reviewed by [1], [2]). One example is the use of signals during territorial defense: a resident animal will use a signal to communicate its intention to chase away a conspecific intruder. If the intruder persists on taking the resident's territory, then the resident will attack. This way of communication supposes that the signal is needed as a pre-requisite for preventing aggressive behavior. Thus, if there is a mismatch between the signal and the behavior and fighting intentions of the signaler, this may lead to a fitness cost for bearers via enhanced aggression by conspecifics [1]–[4]. The mismatch could take place via a non-conventional, novel signal that does not prevent the bearer from fighting. The reason for the fitness cost is that conspecifics would fail to relate the fighting intention of the bearer when assessing the bearer's novel signal (i.e., [5]).

In the context of agonistic interactions, studies supporting aggression by conspecifics and fitness costs that emerge from a mismatch between a signal (i.e., a novel signal) and fighting intentions by the signaler are scarce [1]. One reason for this is that this would require detailed experiments that manipulate either the signal or the subsequent aggression by the signaler, which is not easy. A second reason is that fitness costs are usually indirectly assessed by one or few behavioral responses. In this paper, we have a) experimentally manipulated a signal of fighting ability by producing a novel, non-conventional signal; and b) have measured both ultimate (survival) and underlying proximate (physiological) costs. Since the novel signal implies that the original signal is not properly conveyed, we expected ultimate and proximate costs for the novel signal bearers. As indicated above, these costs would emerge when bearers still use their novel signals but conspecifics fail to recognize it as an indicator of fighting intentions. An analogy to this is the case of aposematic prey studies in which prey with novel colors are preyed upon more often than prey using non-manipulated colors (e.g., [6], [7]).

**Figure 1:**
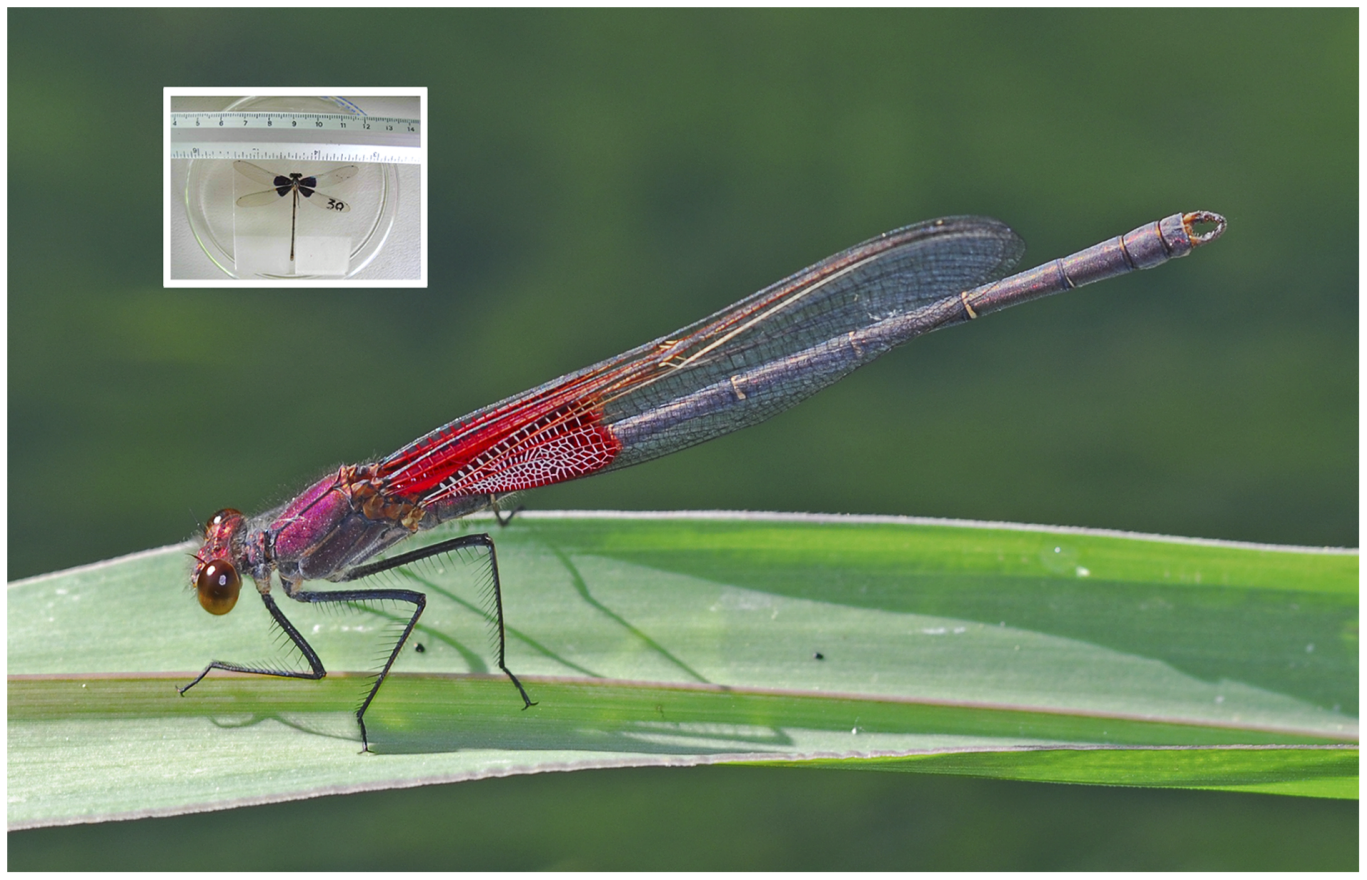
An adult American rubyspot male showing his red wing spot (photo credit Bob Sivinsky). Inset shows how the red spot was modified to blue.

Red coloration is frequently associated with different aspects of fighting ability across animal taxa (e.g., arthropods [8]; fish [9]; reptiles [10]; birds [11] and mammals [12]). One example is the rubyspot damselflies with the red wing spots that male adults bear (Fig. 1). When a non-territorial male is looking for a riverine territory, it wanders through territories and in most cases leaves a place if a territorial male faces and shows the red spots to the former (e.g., [13]). For those rare occasions when a true contest takes place, males with larger spots displace males with smaller spots [14]–[16]. The size of the red spot is selected via intrasexual competition [16] by a long-lasting contest [17] and correlates with several physiological components such as lipid-based fat reserves, muscle mass used for flight and immune ability [14], [17], [18]. These physiological components explain condition and fitness for the following reasons. On one hand, fat reserves and muscle mass are used when a male disputes a territory, which explains their relation with territory tenancy and mating success [19]. On the other hand, both immune ability (in the form of phenoloxidase, a key component in insect immunity) [20] and fat reserves are positively correlated with survival [21], [22]. These different pieces of evidence support the notion for a fighting signal function for wing spot. However, whether the spot red color is used as a first stage to communicate fighting intention by territorial holders, is unclear.

We have used the American rubyspot damselfly, *Hetaerina americana,* as a model to look at the aggressive responses by conspecifics, and physiological and survival costs when using a novel signal. More specifically, we covered the red spot with a blue ink in male territorial holders (Fig. 1). This experiment does not impede the manipulated male to still defend his territory, but allows a mismatch between his fighting intention and the ability to communicate this. We evaluated the behavioral responses of their competing conspecific intruders, field survival of experimental animals (using adequate, modern capture-recapture techniques) [23], [24], and the three key indicators of physiological condition we explained above, as causative of impaired survival: fat reserves, thoracic muscle mass, and immune ability. In line with theory, we predict: (1) compared to control males, males with manipulated wing spots would be more heavily attacked by conspecifics; (2) these increased attacks will correlate with a reduction in survival in experimental males and lower physiological condition.

## Materials and Methods

### Study subject

Recently emerged *H. americana* damselflies take 4–7 days to achieve sexual maturity (all authors' pers. obs). During this time the animals forage to develop muscle mass, accumulate fat body reserves, and develop and fix their red wings patches, which do not subsequently change further after sexual maturity is reached [25], [26]. Once mature, males return to riverine areas where they compete for mating territories [27]. Males may adopt two conditional reproductive tactics (*sensu* in [28]), depending on variables related to their physiological condition (i.e., fat reserves and muscle mass) [13], [14]: territorial and non-territorial. Territorial males are in better energetic (i.e., higher fat reserves and muscle mass) condition and own territories that they defend against conspecifics. Alternatively, non-territorial males are in worse physiological condition, are not able to acquire a space and either fight for one or act as satellites usually in neighboring territories [18], [19]. We only used mature and territorial males. Although we could have used non-territorial males, their extremely low recapture rates (all authors' unpub. data) prevent sound sample sizes. Male maturity criteria were based on the classification of Plaistow and Siva-Jothy [29]. These authors separated adult males into four age-based classes: the first age comprises newly emerged, non-pigmented males, with soft flexible wings and exoskeleton and a zigzag erratic flight. A second category includes males with undamaged flexible wings and thorax, the red wing spot is about to be fully colored, and the main male behavior is foraging. A third age category comprises sexually active males (as judged by their continuous fighting activity on territories), which bear fully developed and conspicuous red wing patches [17]. A last category includes old males, which have abundant pruinescence upon the abdomen and thorax, and opaque, inflexible, and sometimes broken wings [29]. To distinguish territorial from non-territorial males, we carried out behavioral observations. Territorial males were those animals that fought conspecific males to defend a small riparian area (vegetation or rocks in a ratio of 1–3 m^2^) and were not displaced by conspecific males for at least 20 min [14]. Males that did not hold territories were considered non-territorial.

For all experiments we only used territorial and mature males of age class 3. After capture with an insect net, each damselfly was individually marked on the left posterior wing with a unique combination of three digits using a permanent black marker. These numbers are readable at a distance of 3–4 meters. Following marking, individuals were photographed using a digital camera (Nikon P90, 24×). From these pictures, we measured and then averaged the spot area of the four wings to obtain the proportion of the wing covered by the red pigment using Adobe Photoshop (version CS2). After marking, animals were immediately returned to their original capture sites. Manipulation did not last longer than two minutes.

### Study site

Field work was carried out in the Tetlama River, Morelos, Mexico (18° 45′ 55′′N, 99° 14′ 45′′ W) from September to December 2010. For all experiments in this study, we chose three different transects of 200 m length along the river margins, each transect separated by 800 meters. *H. americana* is unusual among odonates in that both sexes spend most of their adult life within a few hundred meters of their mating areas ([16], all authors' pers. obs.), the distance between transects was used to avoid possible migration among transects. The first transect was used to determine territorial behavior, the second transect to determine survival in the field while the last transect was used to determine predation rate (see below). Observations were carried out from 1000 to 1300 hours, the time at which territorial activity is at a maximum (all authors' pers. obs.).All transects were observed under weather conditions favorable for damselfly territorial and mating activity (i.e., full sun or lightly clouded and air temperature in the shade greater than 21°C) [30].

### General procedure of experimental manipulation of wing spot color

Males were randomly assigned to three groups: two control and one experimental groups. Males in the first control group (hereafter, the “non-manipulated” group), were captured, marked, photographed and returned to their original capture site. Males in the second control group (hereafter, the “sham” group) received the same manipulation but their wing-pigmented area was entirely covered with a transparent marker (COPIC sketch®, Colorless Blender 0). Males in the experimental group (hereafter, the “blue” group) had their red wing pigmented area entirely replaced with a blue coloration using a pigment marker (Bic Mark-It®, color deep sea blue; Fig. 1). Blue color was selected because a) its wavelength differs strikingly from the natural red color, b) adult odonates show trichromatic color-specific responses to colors that differ in spectral peaks (i.e., blue, red and green) [31] and c) blue seems completely unfamiliar to the American rubyspot so, when it comes to conspecific interactions between a blue male and intruder males, the latter would perceive a totally non-traditional signal. Although an alternative could have been to make red disappear completely, we were not aware of any methodology that can do this without killing the animal. Furthermore, for the idea of a novel signal, any color would work as long as it differs in wavelength from red and, therefore, perception, by conspecifics.

The above experimental manipulation assumes that conspecifics perceive color different from blue-manipulated males but not from sham and non-manipulated. To test this, we compared three color properties – brightness, red chroma and hue – among experimental groups. On September 7, 2010, we used 16 non-manipulated males, 11 sham males and 13 blue-manipulated (these males are different to those used for the other manipulations) and measured their wing color properties at the site of the spot. Color properties were measured after manipulation from the values of reflectance (*R*), obtained by the light reflected off the measured red wing spot compared with a white standard (Spectralon^TM^), at 10-nm intervals across the range from 360 to 740 nm using a spectrophotometer (MINOLTA CR-200, Konica Sensing Inc., Osaka, Japan). This range was chosen on the basis of previous measurements of red *R* of the spot in the American rubyspot [17]. Brightness (*Br*) was calculated by (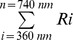), red chroma by (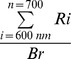) and hue (H) by wavelength where 
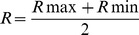
 [32].

### Effects of wing color manipulation on behavior

We captured 120 males and randomly allocated them to the groups mentioned above: non-manipulated (N = 40), sham (N = 40), and blue (N = 40). The study was carried out from November 2 to November 10, 2010. We made focal observations of each resighted marked male for 20 minutes the day after manipulation. The behavioral variables recorded during focal observations were the number of encounters when the territory holder remained in his site after being faced by a conspecific competitor and the number of territorial intrusions. Both variables were evaluated over the total number of encounters in each animal. For this study, we only considered animals that presented any kind of agonistic behavior. Sample sizes of observed animals were as follows: 7 non-manipulated males, 12 sham males and 12 blue.

### Survival cost of lacking red

We captured 278 males and randomly assigned them to the different groups explained above; non-manipulated (N = 92), sham (N = 93) and blue (N = 93). We marked and then recorded the survival of these three groups from September 25 to October 28, 2010. One day after manipulating all individuals we began daily visits to the river to record presence of these animals. With these data, we constructed a binary dataset of daily encounter history for each individual (0 for non-observed and 1 for observed).

We assessed survival using Cormarck-Jolly-Seber models (CJS) [23], as these can dissociate survival (φ) from recapture (p) probabilities [23], [24], [33]. In the construction of models we considered the inclusion of survival and recapture predictors, the experimental groups as a predictor variable and red spot proportion (RSP) as an individual covariate. Capture-recapture models were analyzed using MARK 6.0 software [34] in the framework of maximum likelihood estimation methods [23], [24]. Hence, no frequentist statistical tests (i.e., those based on P values) were performed for analyzing survival in the field (for a similar rationale see [35]). The procedure consisted of creating different models with treatment and RSP as predictors of survival and recapture probabilities. A goodness-of-fit was performed considering φ_(g)_, p_(g)_ as the global model. An important assumption for model selection and parameter estimation is that data should not have severe over-dispersion (i.e., variance ination factor, ĉ>3.0) [23], [24]. The ĉ value was obtained from the global model after performing 1000 bootstrap simulations. A perfect fit of the model would have a ĉ = 1. Greater values are interpreted as over-dispersed data. The global model presented slight over-dispersion (ĉ = 1.107), which is far from serious structural problems or violation of assumptions [23], [24]. Nevertheless, we corrected for over-dispersion using the ĉ obtained from global model, and employed the Akaike Information Criterion for over-dispersion QAICc for model selection and ranking of competing models [36]. Given that there was not a single model with clear support over the others we performed model averaging with the models that represented at least 0.001 of QAICc weight. Hence, we employed model averaging to estimate the effect of experimental manipulation and RSP on survival [36].

### Physiological costs of lacking red

Individuals from transect one (i.e., those used by behavioral observations) were recaptured either 1, 3 or 6 days after manipulation, had their haemolymph extracted (see below), and then stored in ethanol for quantification of fat reserves and muscle mass in the laboratory. The samples sizes for day 1 were: non-manipulated  = 10, sham = 8, blue = 7; the sample sizes for day 3 were non-manipulated  = 8, sham = 4, blue = 14; and the sample sizes for day 6 were non-manipulated  = 15, sham = 9, blue = 7.

### Fat reserves and muscle mass quantification

Fat reserves and muscle mass used for analysis were obtained from the thorax and abdomen since the rest of the body contains insignificant quantities of these compounds [29]. Procedures for measuring fat reserves and muscle mass are as follows. After removing the head and the legs, we dried thoraces and abdomens by placing them in a desiccator (for 24 h) and obtained their dry weight (to nearest 0.1 mg). In order to fat extraction, we placed samples in 100% chloroform for 24 (for similar procedures see [14]) and then were re-desiccated for 24 hours and re-weighed. Fat content was calculated as the difference between initial weight and final weight. After fat extraction, we submerged samples in 0.8 M potassium hydroxide for 48 h to extract the muscle. Again, we dried bodies for 24 hours, re-weighed them and calculated muscle mass as the difference between the pre-potassium hydroxide treatment weight and final weight (for a similar procedure see [17]).

### Phenoloxidase activity in haemolymph

For all treatment groups, we measured phenoloxidase (PO) activity from the haemolymph following the perfusion technique used by González-Santoyo et al, [37]. Since haemolymph samples obtained by perfusion are a mixture of haemolymph and phosphate buffered saline solution in unknown proportions (i.e., some haemolymph samples can be more diluted than others), PO activity was standardized for the total protein mass in the sample. Protein mass was determined using the method of the bicinchoninic acid assay with the PIERCE® protein assay kit. Following determination of protein concentration, PO activity was measured in volumes that contained 20 µg of protein per sample. PO activity in extracted haemolymph was measured by quantifying the formation of dopachrome from L-dihydrophenylalanine (L-DOPA, Sigma) [20].

### Potential negative effects caused by color manipulation

In addition to disrupting the conspecific communication system, we also considered three other negative effects potentially caused by color manipulation that could influence survival, territorial behavior, and physiological condition. The color manipulation itself could cause negative effects via: a) increased wing weights; b) toxic effects of pigment components, and c) predation.

To determine whether wing weight was increased, we painted one anterior wing with either blue or transparent marker and left its corresponding anterior side-pair without manipulation. This was done for eight “blue” and eight “transparent” animals. Each wing was cut at its site of insertion to the body and was weighted twice (in mg) to obtain mean values for the analysis. We compared the wing weight according to treatment.

For testing potential toxic effects of pigments, we kept 10 animals of each group in captivity (i.e., in 5 mL essay tubes with a perching wooden piece and a cap of humid cotton at 26°C) in a 12/12 hrs light-dark regime and with no food. Animals were kept for 36 hours, after which we checked for surviving animals (for similar approach see [38]). Two likely predators of odonates that use visual means are birds and other odonates [39], [40]. Therefore, we carried out an experiment on the third transect to see whether there were differences in predation-based survival between experimental groups. This experiment was conducted from November 15 to November 20, 2010. Seventy five specimens of *H. americana* males were captured and were allowed to die after being placed in a glassine envelope and exposed to direct sunlight. On the following day, these animals were glued to the top of a 30 cm length wooden stick, which was placed in small container filled with water at 8 cm from its base, to avoid predation by ants. Specimens were glued into a natural perching posture at sites where territorial males usually defend territories. A total of 25 triads (each triad consisted of one male for each treatment) were placed along the transect with a separation of 4 m between triads. Animals were left from a total of 4 days, but they were inspected daily for evidence of damage and/or predation. Damaged and/or absent animals were not replaced with new dried specimens. Predation events were inferred by major injury to the specimen (i.e., wings, head, thorax or abdomen completely missing).

### Statistical analysis

To evaluate whether there are differences in color properties among the groups, we used a multivariate analyses of variance (MANOVA) since the color variables we obtained (i.e., brightness, red chrome and hue) are all intercorrelated. Such color properties were used as dependent variables and experimental group was used as an independent variable. Wilk's statistics was used in the analyses and the significance level was set a p<0.05.

As for behavioral and physiological data, given the small samples obtained and that sham and non-manipulated groups did not differ significantly in the proportion of encounters where males remained in their original site after an intruder's invasion (General Linear Models (GLM) binomial: treatment: Likelihood ratio test χ^2^ = 1.9695, *df* = 1, *p* = 0.1605), proportion of aggression received (GLM binomial: treatment : Likelihood ratio test χ^2^ = 1.7015, *df* = 1, *p* = 0.1921), muscle mass (ANOVA: treatment: F_(1,49)_ = 1.2097, *p* = 0.2768), fat reserves (ANOVA: treatment: F_(1,50)_ = 0.7621, *p* = 0.3808) and PO activity (ANOVA: treatment: F_(1,51)_ = 0.1388, *p* = 0.711), we pooled them into a single group now called control.

To assess the effect of red spot manipulation on territorial behavior, all statistical tests included experimental treatment and RSP as predictor variables. For each response variable (i.e., proportion of encounters when territorial males remained in their original site and proportion of aggression received) we fitted GLMs of the binomial family (with logit link function). When residual models were over-dispersed we used a quasi-binomial correction [41]. In models with over-dispersion, we used Likelihood Ratio Test (LRT) for obtaining the simplest appropriate model. LRT is frequently used to determine whether or not data support a full model over a reduced model [35]. We searched for the simplest model by beginning with a global model that contained the most parameters and then compared it to a simpler model with one fewer parameter. The global model was Group:RSP, following comparisons by Group+RSP and finally with Group. When LRT of the most complex model was significantly greater than the simplest model (with a *p*-value<0.05) then the complex model was chosen, and vice versa. Selection of the most complex model indicates that the benefit of the improved model fit outweighs the cost of higher model complexity [35].

We checked for the presence of influential observations in each selected model by measuring the Cook's distance of each observation (values greater than 1 are considered influential) [42]. However, we did not detect any outliers in any model tested.

To evaluate the effect of wing color manipulation on muscle mass, fat reserves, and PO activity, we fit linear models (LM), now adding the number of days between wing manipulation and recapture as an additional predictor variable. The best model (with and without interactions) was chosen based on the lowest AIC value [43]. When the new predictor variable (i.e., RD) significantly influenced any response variable we then performed a pairwise comparison between groups using a t-test with pooled standard deviation [41]. We used Fligner-Killeen tests to assess homogeneity of variance in the residuals and visual observations to assess normality [41].

For weight differences in wing pairs (one of them covered with ink), we used paired *t* tests. For toxic effects of blue and predation rate, the rates of survival among individuals in each treatment group were compared using χ^2^ tests. Analyses were made in R software (R Development Core Team 2009, v.2.1.3.2) and SPSS (v. 15.0).

Animals were collected using a permit from the Secretary of Environment and Natural Resources (SEMARNAT) issued to AC-A. However, our study does not involve endangered or protected species. During manipulation, every effort was made to reduce animal suffering.

## Results

### Color properties among experimental groups

There was a statistically significant difference in the three color properties based on experimental groups (MANOVA: *F*_2,37_ = 17.039, *P*<0.001; Wilk's Λ = 0.165). However, blue animals showed significant differences in brightness (ANOVA: *F*_2,37_ = 17.34, *P<*0.001), chroma (ANOVA: *F*_2,37_ = 32.747, *P<*0.001) and hue (ANOVA: *F*_2,37_ = 38.155, *P<*0.001) when compared with non-manipulated (brightness: *t*_2,37_ = 5.478, *P*<0.001; chroma: *t*_2,37_ = 6.717, *P*<0.001; hue: *t*_2,37_ = 7.363, *P*<0.001) and sham animals (brightness: *t*_2,37_ = 4.624, *p*<0.001; chroma: *t*_2,37_ = 7.280, *P*<0.001; hue: *t*_2,37_ = 7.766, *P*<0.001), but there were no differences between non-manipulated and sham males (brightness: *t*_2,37_ = −0.385, *P* = 0.921; chroma: *t*_2,37_ = 1.211, *P* = 0.454; hue: *t*_2,37_ = 18.33, *P* = 0.518).

### Effects of wing color manipulation on behavior

**Figure 2:**
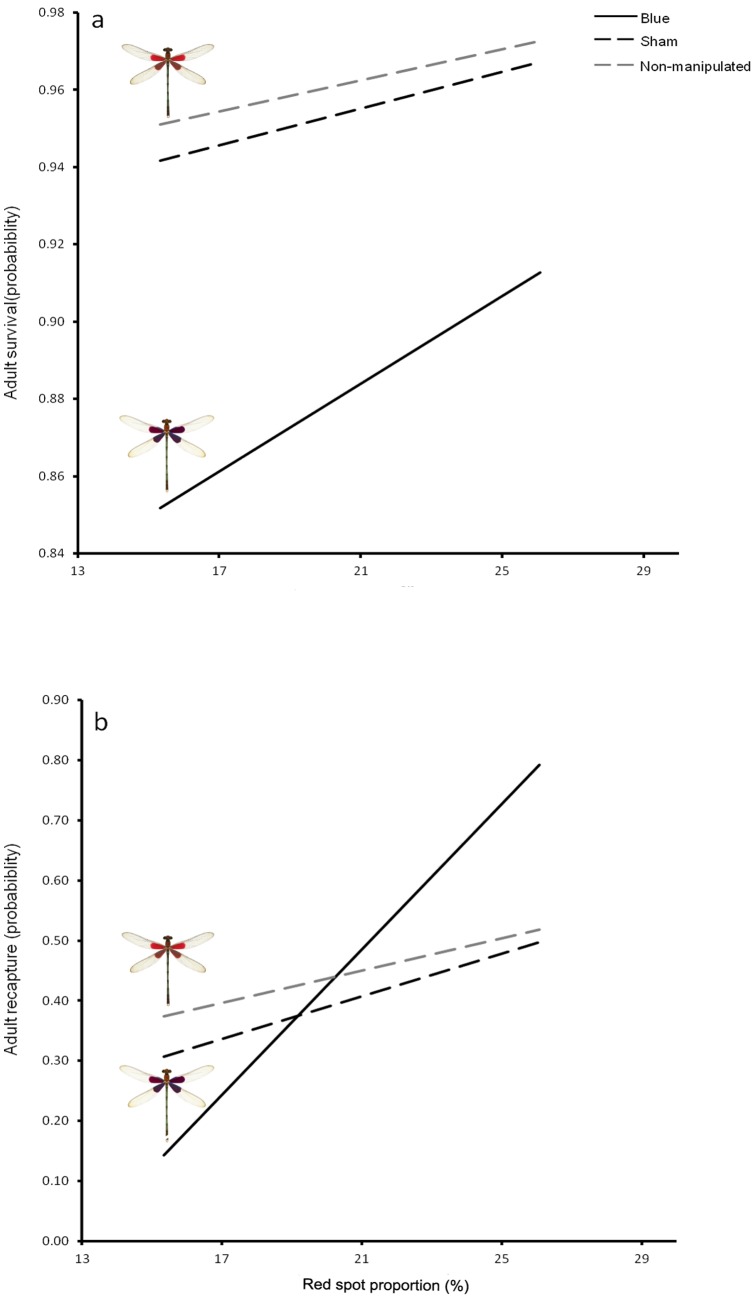
The proportion of encounters when the holder remained in the site after an intrusion according to experimental group. Boxes represent first, second and third quartiles; whiskers are the sample maximum and minimum observations; sample sizes are shown above each plot.

For the number of encounters where the holder remained in its site after an intruder's invasion, the simplest model selected only considered experimental group as predictor variable. According to this, group had a significant effect on the probability of remaining on a territory following territorial intrusions (GLM: Group: LRT = 14.316, *df* = 1, *P* = 0.031; Table 1). Blue males were less likely to remain on defended sites following territorial intrusions (38.1±37.9 %) than control males (66.0±34.4 %; Table 2; Fig. 2). In proportion of aggression received, the simplest model selected did not include any predictor variable (i.e., the addition of covariates did not improve model fit; Table 1). That is, all groups were similarly attacked. Finally, there were no differences in the total number of encounters during the period (20 minutes) of focal observations (blue: 6.084±1.12; control: 5.210±1.436; *F*_1,29_ = 0.369, *P* = 0.548).

**Table 1:** Comparisons between paired models by the Likelihood Ratio Test for (a) proportion of encounters that the holder remained after an intrusion and (b) the proportion of territorial intrusions divided by the total number of encounters.

Encounters remaining	*Compared with*	LRT	Df	P
∼Group+RSP+Group:RSP	∼Group+RSP	93.38	1	0.448
∼Group+RSP	∼Group	95.14	1	0.798
∼**Group**	∼1	**95.33**	**1**	**0.031** *

Aggression received	*Compared with*			
∼ Group+RSP+Group:RSP	∼Group+RSP	87.67	1	0.902
∼Group+RSP	∼Group	87.71	1	0.869
∼Group	∼**1**	87.77	1	0.318

The model comparison started with the most complex (i.e. the highest number of parameters) model versus the model with one parameter less until the simplest appropriated model is found.

The simplest appropriated models are in bold.

Significance of compared model with P<0.05.

“∼1” without predictor variable.

**Table 2:** Descriptive statistics (mean ± SD) for physiological and behavioral variables of experimental groups according to their recording time.

Physiology	1day		3 days		6 days	
	Control (15)	Blue (7)	Control (12)	Blue (14)	Control (24)	Blue(7)
Fat reserves (mg)	0.29±0.27	0.19±0.19	0.43±0.29	0.25±0.25	0.19±0.17	0.15±0.20
Muscle mass (mg)	2.10±0.80	2.00±0.90	1.83±1.79	1.09±0.85	2.03±1.20	1.10±1.10
PO activity(OD/µg protein)	0.55±0.31	0.40±0.34	0.76±0.21	0.76±0.19	0.80±0.23	0.78±0.22

Behavior	Control (12)	Blue (19)		
Encounters remaining (%)	66.03±34.33	38.14±37.90		
Aggression received (%)	50.54±36.21	67.00±35.62		

### Survival cost of lacking red

**Figure 3:**
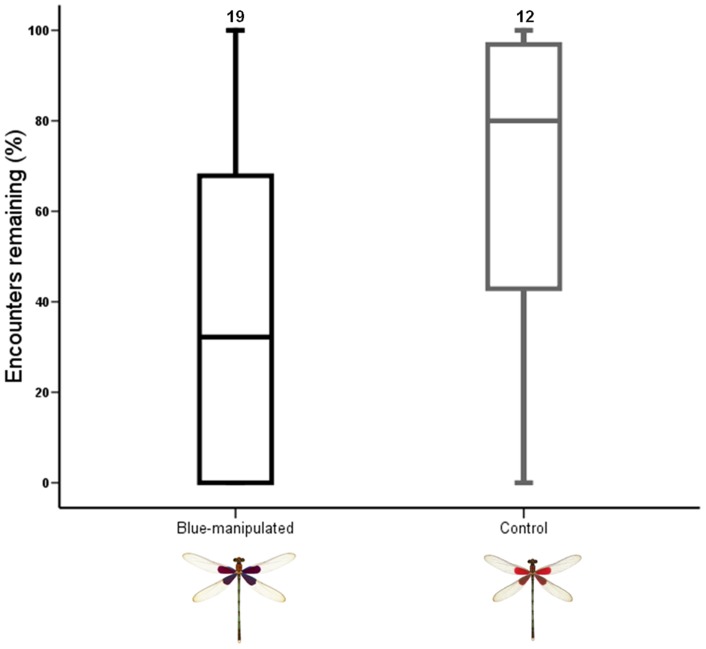
Predicted survival (a) and recapture (b) values according to red spot proportion and experimental group.

Cormarck-Jolly-Seber models indicated that survival rates were best predicted by a model that included the main effects of both treatment and RSP. Recapture rates were best predicted by a model that included the interaction between treatment and wing pigmentation, as well as the main effects of each (Table 3). Blue males had lower survival compared to sham and non-manipulated males, but there were no differences in survival between sham and non-manipulated males (Table 4). Additionally, in all groups males with larger wing spot survived for longer as indicated by the slopes in blue (β = 0.567, CI 95 %: 0.524 to 0.609), sham (β = 0.237, CI 95 %: 0.219 to 0.254) and non-manipulated males (β = 0.205, CI 95 %: 0.189 to 0.220). Sham and non-manipulated males did not differ in their slopes (Fig. 3a). The same relation was also observed for recapture probability in blue (β = 0.786, C.I 95 %: 0.688 to 0.884), non-manipulated (β = 0.169, C.I 95 %: 0.081 to 0.257) and sham males (β = 0.035, C.I 95 %: −0.041 to 0.111; Fig. 3b).

**Table 3:** Selection of higher supported models (AICc weight) whose survival (φ) and recapture (p) parameters include wing spot proportion (RSP) and experimental groups.

Model						
Survival	Recapture	AICc	ΔAICc	AICc weight	Parameters	Deviance
φ_(group+RSP)_	p_(group*RSP)_	2894.401	0.000	0.249	10	2874.17
φ_(group * RSP)_	p_(group*RSP)_	2894.444	0.043	0.243	12	2870.11
φ_(group* RSP)_	p_(group)_	2895.081	0.680	0.177	8	2878.93
φ_(group* RSP)_	p_(group+RSP)_	2895.551	1.150	0.140	10	2875.32
φ_(group+ RSP)_	p_(RSP)_	2896.083	1.682	0.107	6	2883.99
φ_(group+ RSP)_	p_(group+RSP)_	2896.610	2.207	0.082	8	2880.45
φ_(group* RSP)_	p_(.)_	2904.701	10.300	0.001	7	2890.58

**Table 4:** Daily estimation of survival and recapture probabilities according to experimental group. Estimation was done according to the weight of the highest supported models.

					95% confidence interval	
	Treatment	Parameter	Estimate	Standard error	Lower	Upper
Survival	Blue	φ_B_	0.871	0.017	0.835	0.900
	Sham	φ_S_	0.952	0.007	0.936	0.964
	Non-manipulated	φ_N_	0.960	0.006	0.946	0.971
Recapture	Blue	p_B_	0.401	0.033	0.339	0.468
	Sham	p_s_	0.388	0.023	0.343	0.434
	Non-manipulated	p_N_	0.424	0.022	0.383	0.467

### Physiological cost of lacking red

**Figure 4:**
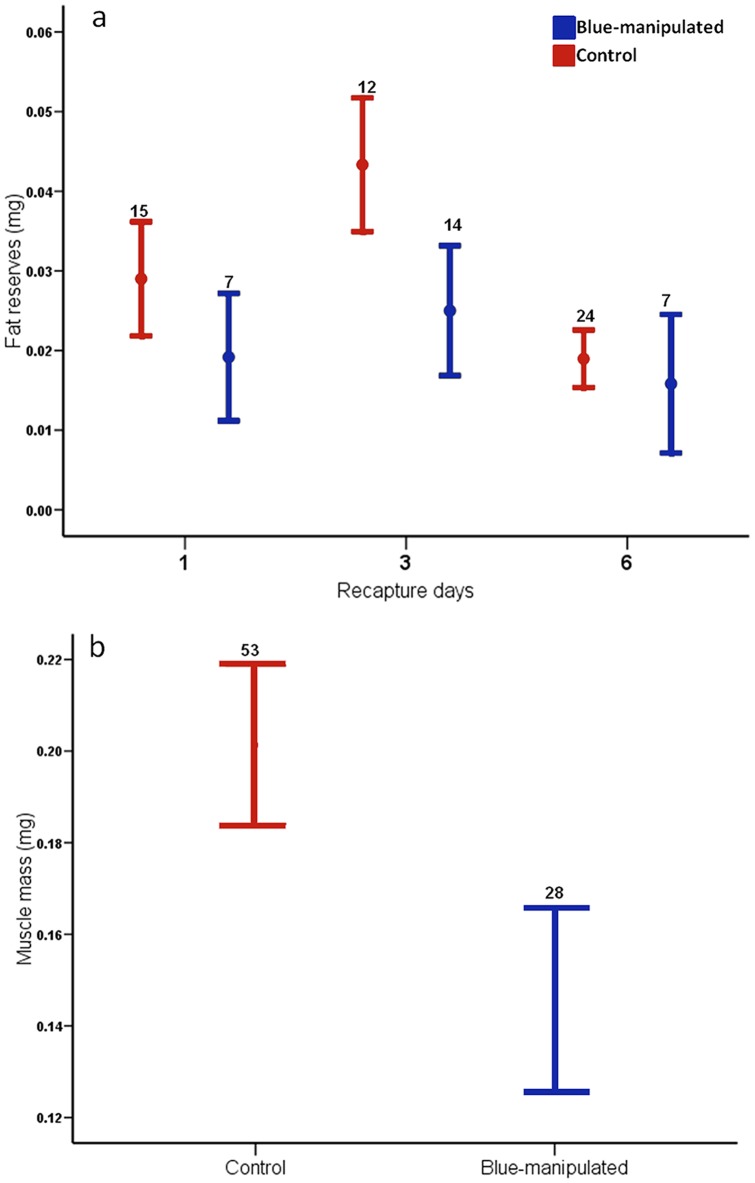
Fat reserves in relation to recapture day (a), and muscle mass according to experimental group (b). Lines show mean ± Standard errors. Sample sizes are shown above each plot.

Fat reserves were affected by group and recapture day (RD) but not by RSP (*F*_2,69_ = 3.158, *P* = 0.030; Table 5). Six days after manipulation, fat reserves were lower than after 3 days (*t*_55_ = −2.864, *P*<0.010; Table 6), but did not differ one day after manipulation (*t*_54_ = −1.309, *P* = 0.195) or between days one and three (*t*_49_ = 1.468, *P* = 0.147; Table 2; Fig. 4a). Fligner-Killeen tests confirmed homogeneity of variance (χ^2^ = 4.5608, *df* = 2, *P* = 0.102). Muscle mass was only affected by group (*F*_1,74_ = 4.740, *P* = 0.032; Table 5; Fig. 4b): blue males had lower muscle mass than control males (Table 5). Fligner-Killeen tests confirmed homogeneity of variance (χ^2^ = 0.014, *df* = 1, *P* = 0.906).

**Table 5:** Model selection for each physiological variable obtained from the lowest AIC value.

Variable	Models	Df	Deviance	AIC
Fat reserves				
	G+RD+RSP+G:RD+G:RSP+RD:RSP**+G:RD:RSP**			−1208.19
	G+RD+RSP+ G:RSP+RD:RSP**+G:RD**	2	1.1e-07	−1209.84
	G+RD+RSP+G:RSP**+RD:RSP**	2	5.1e-08	−1212.78
	G+RD+RSP**+G:RSP**	2	0.4e-07	−1213.92
	G+RD**+RSP**	1	7.5e-08	−1214.45
	G+RD	1	1.9e-08	−1216.06*
Muscle mass				
	G+RD+RSP+G:RD+G:RSP+RD:RSP**+G:RSP:RD**			−1013.21
	G+RD+RSP+ G:RSP+RD:RSP**+G:RD**	2	1.1e-07	−1017.11
	G+RD+RSP+ RD:RSP**+G:RSP**	2	9.1e-05	−1019.69
	G+RD+RSP**+RD:RSP**	1	9.2e-05	−1021.42
	G+ RSP**+ RD**	2	9.5e-05	−1022.95
	G**+RSP**	2	9.7e-05	−1024.75
	G	1	1.0e-04	−1024.93*
PO Activity				
	G+RD+RSP+G:RD+G:RSP+RD:RSP+**G:RSP:RD**			−204.11
	G+RD+RSP+G:RSP+RD:RSP**+G:RD**	2	0.033	−207.44
	G+RD+RSP+G:RSP+RD:RSP	2	0.056	−210.34*

Model selection started with the global model that contained Group (G), recapture days (RD), wing spot proportion (RSP) and their interactions (:).

Removed parameters for the consequent comparison model are in bold.

Selected model with lowest AIC value.

**Table 6:** Main effects of selected linear models over physiological variables.

Variable	Model selected	Estimate	SE	T	P
Fat load	Group+RD				
	Group				0.084
	**RD**				0.**047** *
	Day 1 vs Day 3	−1.063e-04	7.25e-05	1.468	0.147
	Day 1 vs Day 6	−8.783e-05	6.71e-05	−1.309	0.195
	Day 3 vs Day 6	−1.942e-04	6.79e-05	−2.864	**0.006** *
Muscle mass	Group	−6e-04	−3e-04	−2.178	**0.032** *
PO activity	Group+RD+RSP+G: RSP+RD:RSP				
	Group				0.510
	G:RSP				0.292
	RD:RSP				0.072
	**RSP**				**0.020** *
	**RD**				**0.000** *
	Day 1 vs Day 3	1.854	0.737	2.518	**0.010** *
	Day 1 vs Day 6	0.923	0.463	1.992	**0.050** *
	Day 3 vs Day 6	−0.931	0.758	−1.230	0.220

Parameters with P<0.05 are in bold.

Significance of the main effects at *p*<0.05.

PO activity was best explained by the model that includes all explanatory variables and the interactions of red spot proportion (RSP) with group and recapture day (RD) (*F*_7,68_ = 4.635, *P*<0.001; Tables 5 and 6). In all groups, PO activity was lower 1 day after manipulation than after 3 days (*t*_49_ = 4.635, *P*<0.001) and 6 days (*t*_54_ = 4.043, *P*<0.001). There were no differences between 3 and 6 days after manipulation (*t*_55_ = 0.116, *P* = 0.998; Table 4). RSP also showed a significant effect in PO activity (β = 4.815, *t*_68_ = 2.316, *P* = 0.023; Table 5) but not in the interaction with groups. Fligner-Killeen confirmed homogeneity of variance (χ^2^ = 3.749, *df* = 2, *P* = 0.154).

### Potential negative effects caused by color manipulation

There was no difference in weight between pairs of anterior wings (one painted and one not painted) either for blue (paired *t* test, *t_7_* = 0.428, *P* = 0.681; weight added in each pair of wings: 0.045±0.056 mg) or clear manipulation (paired *t* test, *t_7_* = 1.374, *P = *0.21; weight added in each pair of wings: 0.054±0.043 mg). We did not detect any toxic effects of pigment components since numbers of surviving animals held in captivity among treatments were not significantly different (χ^2^ = 0.765, N = 30, *df* = 2, *P* = 0.681). Four days after dead animals were placed in triads for predation testing, there were no significant differences in the rate of removal of animals across treatment groups (number of individuals removed: blue = 5, sham = 6, non-manipulated  = 5; GLM: binomial:Treatment: LRT χ^2^ = 0.101, *df* = 2, N = 75, *P* = 0.950).

## Discussion and Conclusions

Being unable to detect potential negative effects of our manipulation, we have shown that males with a non-conventional, novel signal (a blue spot; Fig. 1) had a reduced survival and deteriorated physiological condition but were not more attacked compared to control males. In what follows we will try to establish a functional connection between survival, behavior and physiology. That a reduced survival is not explained by aggressive attacks by conspecifics contradicts theory [1], [3]. Note, however, that although all groups were similarly attacked, blue males were less likely to remain in their territories. Thus, other than the number of aggressive attacks, there may be other unmeasured behavioral variables that explain why blue males lost their defended sites. One case is contest duration: contests with blue males may have lasted longer than with other males, which led the blue males to become more energetically exhausted. On the other hand, the reduced survival in blue males may be explained by a negative effect on muscle mass. Fat reserves followed the same trend despite being non-significant. Although both, muscle mass and fat reserves are predictors of territorial roles (territorial and non-territorial behavior) and fighting success in the American rubyspot [17], [19], their relation is complex [44]. For example, adult fat reserves seem more immediately affected by long-lasting contests compared to muscle mass, although after fighting males usually end up with a considerable reduction in muscle mass [17], [39]. Thus, perhaps after a number of long-lasting fights for a number of days, blue individuals were no longer able to defend their sites because their muscle mass tissue was too deteriorated to sustain flight and compete for territories. This may have occurred even for those blue males that attempted to re-gain a territory (as American rubyspot males can switch from territorial to non-territorial behavior and viceversa [44]). However, whether such switch took place, we do not know.

One may wonder what conspecifics perceived when males displayed the blue spot. Encounters between conspecific males are very common in the American rubyspot. Non-territorial males frequently fly over territories and, after facing an opponent for a fraction of second, leave immediately. This behavior has been interpreted as a respect for the “residency” asymmetry in calopterygids [45]. “True fights” occurs only if territory availability becomes too scarce [29], [46]. We believe that one function of short-lasting encounters by non-territorial males is to “check” whether a territory is available, which is assessed via by the territorial male's red spot. This level of communication may not only be used with conspecific males but also males, as interspecific territorial competition are common in the *Hetaerina* genus [30]. We cannot entirely discount the possibility that blue males were mistakenly identified given that even less drastic wing color manipulation can cause *Hetaerina* males to be classified as heterospecifics [30]. However, we feel it is highly unlikely as the American rubyspot is the only *Hetaerina* species in the study site.

Previous studies in birds have indicated that aspects of red coloration (i.e., redness) may also be a signal component along with signal size [47], [48]. These studies have found that since redness is an indicator of male condition [49], [50], this color aspect may be taken as a general indicator of condition. In relation to our study subject, the better the diet a larva has, the larger the spot in the corresponding adult [51]. However, when true fightings take place, spot chroma, hue and brightness do not predict contest outcome but wing spot size does [15], [18]. In fact, these three aspects are relatively invariable thus they are not predicted by male diet or territorial status [15]. Spot size, however, does not seem selected during that first level of communicating residency according to our observations of aggressions. This suggests that, at this first step of communication, a large spot size is not needed but, possibly, the red nature only.

Our result that spot size correlates with survival supports a similar finding in the same species [52]. Interestingly, although experimentally increasing the size of the spot enhances the ability to hold territories [16], it negatively affects survival [53]. These previous results and what we have shown here are complimentary: while this previous evidence indicates an ecological cost of displaying the signal, our results indicate that a modified color version of a signal implies a social cost. Thus, it seems that in American rubyspots the communication system is reliable given that levels of signal intensity accurately accompany levels of signaler quality [16] and that several costs counteract an inadequate expression of the fighting signal.

In summary, theoretical studies for the evolution of signals indicate a match between signal design and fighting intentions [1], [2], [3]. Although our results do not provide strong evidence towards higher aggression for males with a novel signal that does not communicate fighting intention, they lend support to a survival cost mediated by physiological mechanisms.
